# Plant Growth‐Promoting Rhizobacteria Colonize Δ^9^ ‐Tetrahydrocannabinolic Acid Drug‐Type *Cannabis sativa* L. Roots and Modulate Cannabinoid Metabolism

**DOI:** 10.1111/ppl.70756

**Published:** 2026-01-24

**Authors:** Francesco Tonolo, Bobbie Sewalt, Klaas Vrieling, Young Hae Choi

**Affiliations:** ^1^ Aboveground‐Belowground Interaction Group, Plant Cluster, Institute of Biology Leiden University Leiden the Netherlands; ^2^ Natural Products Laboratory, Institute of Biology Leiden University Leiden the Netherlands

**Keywords:** bacillus, biostimulants, Cannabis sativa L., PGPR, pseudomonas

## Abstract

Plant growth‐promoting rhizobacteria (PGPR) establish beneficial associations with plants, enhancing nutrient uptake, growth, and stress tolerance. *Cannabis sativa* L., a medicinal plant producing over 300 specialized metabolites with relevant medicinal properties, remains underexplored for PGPR influence on its metabolism. This study assessed the ability of four PGPR taxa: *Bacillus*, *Pseudomonas*, *Flavobacterium*, and *Burkholderia* to colonize roots and modulate cannabinoid metabolism. Two Δ^9^‐tetrahydrocannabinolic acid (THCA) drug‐type *C. sativa* cultivars, Amnesia Haze and Gorilla Glue, were tested. Plants grown hydroponically were inoculated under controlled conditions. Root colonization was confirmed via endophyte‐specific assays. Phenotypic analyses revealed no effects on plant phenotype, while chemical analyses revealed a response shared across taxa and cultivars. Bacterial inoculation increased the precursor cannabinoid Cannabigerolic acid (CBGA) concentration significantly by +27.37% while reducing Δ^9^‐tetrahydrocannabinol (Δ^9^‐THC) by −15.76%. The CBGA/THCA and THCA/CBDA ratios shifted significantly, indicating a favored CBGA accumulation and CBDA production, respectively. PGPR treatments reduced in vivo and post‐harvest decarboxylation of THCA into Δ^9^‐THC, preserving the acidic cannabinoid profile. Under a standardized, soilless hydroponic regimen with a single shared reservoir and identical fertigation across groups, PGPR colonization was associated with shifts in cannabinoid metabolism and reduced decarboxylation. This study demonstrates that PGPR can influence the specialized metabolism of high‐THCA *C. sativa* , offering insights into sustainable cultivation and pharmaceutical exploitation of this relevant medicinal plant species.

## 1 Introduction

Plants and bacteria share a long history that spans over a 100 million years. Since then, they have co‐evolved to form complex relationships that facilitate the survival of both. One of the most intriguing associations is the symbiotic relationship between rhizobacteria and plants (Lyu et al. 2021). Rhizobacteria are a group of bacteria inhabiting the soil surrounding plant roots (Hiltner 1904). The symbiotic relationship between bacteria and plants pivots mainly, but not only, around the exchange of nutrients. It has been estimated that up to 10% of the plant photosynthates is redirected into the soil as root exudates comprising sugars, amino acids, organic acids, and specialized metabolites (Jones et al. 2004, 2009). These organic exudates feed the bacteria, which in turn provide a series of services for the plants that usually result in fitness promotion. Some of these bacteria even have plant growth promotion effects, and therefore, they have been called plant growth‐promoting rhizobacteria (PGPR) (Kloepper et al. 1980).

PGPRs can promote growth in several ways. Most notably, they can increase the availability of minerals by fixing atmospheric nitrogen. They can help to increase the plants' uptake of several nutrients such as Ca, K, Fe, Cu, Mn, Zn, and particularly phosphorus through solubilization and chelation. PGPRs can also produce signaling molecules mediating plant‐microbe interactions, such as plant hormones like auxins, cytokinins, gibberellins, and volatile organic compounds promoting plant growth. PGPRs can also positively influence plant growth by protecting plants against pathogens through competition for nutrients, minerals and colonization sites, production of antibiotics, and by promoting the induced systemic resistance (ISR) (Pérez‐Montaño et al. 2014). Interestingly, it has also been documented that PGPRs can influence and modulate the specialized metabolism of several aromatic and medicinal plants (Banchio et al. 2009; Del Rosario Cappellari et al. 2013, 2019). For example, *Azospirillum brasilense* , when inoculated on hydroponically grown sweet basil ( *Ocimum basilicum* L.), induced changes in alkaloid, phenylpropanoid and terpene metabolism in a cultivar‐dependent manner (Kolega et al. 2020).

Surprisingly, although *Cannabis sativa* L. produces a suite of specialized metabolites, among which are 150 cannabinoids, 120 terpenoids, 42 phenolics, 34 flavonoids, and two alkaloids (Hanuš et al. 2016; Pollastro et al. 2018; Radwan et al. 2021) that have a large number of potential medicinal applications (Andre et al. 2016), the effects of PGPRs on its metabolism are still unclear. Cannabinoids are the compounds for which *C. sativa* is most well known. The most important are cannabidiolic acid (CBDA), Δ^9^‐tetrahydrocannabinolic acid (THCA) and cannabigerolic acid (CBGA). The latter is synthesized by condensation of olivetic acid with geranyl pyrophosphate; it is the first cannabinoid of the pathway, and the precursor for the production of THCA and CBDA, which is carried out by the respective synthases (Zirpel et al. 2018). The pharmaceutically active cannabinoids are obtained by exposing cannabinoid acids to heat or radiation, leading to decarboxylation into Δ^9^‐tetrahydrocannabinol (Δ^9^‐THC) and cannabidiol (CBD) (Filer 2022).

Several studies have investigated the presence, distribution, and diversity of bacteria and fungi in healthy (Kusari et al. 2012; Gautam et al. 2013; Winston et al. 2014; Afzal et al. 2015; Scott et al. 2018; Barnett et al. 2020; Comeau et al. 2020; Ahmed et al. 2021; Wei et al. 2021; Punja and Scott 2023) and diseased *C. sativa* (Punja et al. 2019). Observations of the *C. sativa* microbiome revealed that a wide range of bacterial as well as fungal endophytes are present in roots, leaves, petioles, stems, and flower tissues in a cultivar/spatiotemporal/substrate‐dependent manner, demonstrating the possibility of colonization by known beneficial bacteria. Although only a few studies have assessed the potential growth promotion effects of PGPRs on the phenotype of *C. sativa* (Conant et al. 2017; Pagnani et al. 2018; Balthazar et al. 2020; Comeau et al. 2021; Lyu et al. 2022; Ahmed et al. 2023; Lyu et al. 2023), it appears that PGPRs generally have a positive influence on several growth parameters. On the other hand, four studies have investigated the potential use of PGPRs as biocontrol agents against *C. sativa* pathogens with promising results (Balthazar et al. 2020; Scott and Punja 2020; Balthazar, Novinscak, et al. 2022; Balthazar, St‐Onge, et al. 2022).

Meanwhile, four studies have assessed the effects of PGPRs on the cannabinoid production of this medicinal plant species (Pagnani et al. 2018; Ahmed et al. 2023; Lyu et al. 2023; Tanney et al. 2023). Unfortunately, Pagnani et al. (2018) measured only Δ^9^‐THC and CBD without converting all CBDA and THCA into CBD and Δ^9^‐THC. Lyu et al. (2023), while Tanney et al. (2023) presented contradicting results and Ahmed et al. (2023) failed to observe the inoculated microbes in their sequencing datasets. Specifically, Lyu et al. (2023) studied the effects of *Bacillus*, *Mucilaginibacter*, and *Pseudomonas* on a single genotype of the drug‐type cultivar CBD Kush, which presents a balanced THCA and CBDA cannabinoid profile, and they found an increase in THCA and CBDA upon inoculations in the vegetative stage. In a follow‐up study (Tanney et al. 2023) using the same bacterial strains and plant genotype and grown in soil but under two fertilization regimes, the authors showed that none of the cannabinoids measured differed significantly from the control at the recommended fertilization levels. The two main cannabinoids, THCA and CBDA, although not significantly, were found to decrease in all the bacterial treatments compared to control conditions, opposite to their previous experiment (Lyu et al. 2023). Ahmed et al. (2023) inoculated clonally propagated plants presenting different chemotypes with three different microbial consortia. Unfortunately, as stated by the authors, none of the inoculated microbes were found in their soil and roots sequencing datasets. At the same time, the metagenomic analysis revealed that several known *C. sativa* pathogens, such as *Fusarium concentricum*, *Fusarium oxysporum*, and *Fusarium solani*, were detected in the roots with important implications for the experiment outcome (Gwinn et al. 2022).

Despite these previous findings, the effects on cannabinoid metabolism due to PGPR inoculation remain unclear. Moreover, as we gradually understand *C. sativa* and how the cannabinoid metabolism reacts to different biotic stimuli, it appears that nutrients can influence their accumulation (Bernstein et al. 2019; Shiponi and Bernstein 2021; Saloner and Bernstein 2022a, 2022b; Llewellyn et al. 2023). For these reasons, it is difficult to tease apart if the potential effects of PGPRs on cannabinoid metabolism are induced directly by bacterial colonization or indirectly by modulation of nutrient availability in the soil.

Therefore, we designed an experiment to evaluate the ability of PGPRs from four distantly related taxa to colonize the tissues of *C. sativa* and measure the effects on cannabinoid profile while, at the same time, disentangling the relationship by ruling out the potential effect of nutrient availability and influence of other organisms. In order to achieve this, we firstly chose the PGPR strains based on their ability to penetrate, colonize, and influence the growth and metabolism of other crops (Van Der Voort et al. 2015; Carrión et al. 2018, 2019; Berlanga‐Clavero et al. 2022). Secondly, we chose two widely used commercial drug‐type THCA‐dominant cultivars and seeds were used for the experiment instead of a single clonally propagated genotype, in order to study cultivar‐level responses under realistic biological variance and therefore provide broader relevancy and applicability. Thirdly, plants were grown in a soilless hydroponic setting. Compared to regular soil, the Rockwool substrate is initially sterile and is progressively colonized by algae, fungi, and bacteria. The simpler matrix allows us to study the plant–microorganism interaction with fewer unwanted or complex influences and allows us to rule out the effect of nutrient availability as optimally balanced mineral fertilization for *C. sativa* growth is typically provided in excess multiple times a day. While this setup allows for a rhizosphere easier to study, it is important to note that the effects of bacteria on plants grown in soil might still be different from a hydroponic soilless setting.

Specifically, we want to answer the following questions: Can the four PGPR strains penetrate and colonize the root system in a soilless hydroponic setting? Does PGPR colonization influence the plant growth of high THCA *C. sativa* cultivars? Does PGPR colonization influence the cannabinoid metabolism of high THCA *C. sativa* cultivars? Are the observed effects on *C. sativa* phenotype and cannabinoid metabolism PGPR‐ or cultivar‐dependent?

## 2 Materials and Methods

### 2.1 Bacterial Growth Conditions

Bacteria of each of the genera under study, *Bacillus* (Berlanga‐Clavero et al. 2022), *Pseudomonas* (Van Der Voort et al. 2015), *Flavobacterium* (Carrión et al. 2019), and *Burkholderia* (Carrión et al. 2018), possessing antibiotic resistance, were obtained from Dr. Victor Carrión Bravo (Institute of Biology, Leiden University/Malaga University). Bacterial strains were incubated in liquid LB media (Sigma–Aldrich) at room temperature. During the log phase, samples were measured with Eppendorf BioSpectrometer basic, and dilution with fresh LB media was carried out to reach an inoculation concentration of 10^8^ CFU/mL. Inoculation was carried out immediately after dilution.

### 2.2 Endophyte Inoculation and Methodology

Bacterial endophyte inoculation was carried out twice after plant transplantation into the 1‐L Rockwool cubes (Rockwool) and inoculation was carried out at day 21 and once more at day 35 after germination. The inoculation was performed by pipetting 2 mL of bacterial solution at a concentration of 10^8^ CFU/mL directly on the Rockwool substrate at the base of the stem. Control plants were inoculated with a sterile mock solution composed of the same media in which the bacteria were propagated. The automated fertigation system was turned off for 4 days after inoculation to avoid flushing away the bacteria from the substrate and allow for the colonization of the root system. During this period, none of the plants wilted. Several steps were taken to conduct the experiment in a semi‐sterile environment and avoid cross‐contamination between treatments. Briefly, the Rockwool cubes were placed on an upside‐down dish to raise them off the table and avoid possible contact of the cubes or roots with the excess nutrient solution drained from other cubes. Rockwool blocks were positioned on stainless‐steel tables, sterilized with 3% (v/v) hydrogen peroxide solution every week to prevent the possible buildup of bacteria and algae. The reservoir containing the nutrient solution was kept closed and in darkness to avoid the possible growth of algae and bacteria. The nutrient solution was prepared starting from reverse osmosis deionized water and following typical aseptic tissue culture techniques so as not to contaminate the reservoir. The top of the Rockwool cubes was covered with a thick black plastic sheet to avoid the growth of algae and fungi, leaving only a small hole for the plant stem to exit the cube. All the equipment used was sterilized thoroughly with 0.5% (v/v) NaClO solution and rinsed with demineralized water before the start of the experiment. The room was cleaned with 0.5% (v/v) NaClO solution before the start of the experiment and weekly thereafter. Plants and tables were never touched after the initial setup. The drain solution collected in reservoirs under the tables was first sterilized with 0.5% (v/v) NaClO solution before being discarded. Only two researchers working on the experiment could access the walk‐in growth chamber, which was permanently closed. For each of the four bacterial strains and the control, eight replicate plants were used for each *Cannabis sativa* cultivar (Table S1).

### 2.3 Plant Material and Growth Conditions

*C. sativa* seeds of two common feminized commercial drug‐type cultivars were purchased from Snorkel Spain SLU for the experiment. The cultivars were chosen for their high cannabinoid productivity, relatively low chemical variability and different genetic background in order to study cultivar‐level responses under realistic biological variance. Both cultivars were THCA dominant and presented high total cannabinoid concentrations, 24.03% ± 3.46% for cultivar Amnesia Haze and 19.29% ± 1.70% for cultivar Gorilla Glue (Fernandes et al. 2023). Plant growth was carried out following standard agricultural practices for producing *C. sativa* inflorescences, as previously described by Pagnani et al. (2018). To minimize possible variation in the treatment of individual plants, the nutrient solution was prepared and administered to all plants from the same reservoir at the same time. Therefore, the concentration and fertigation regime were the same for all plants. Additionally, plants were placed in a growth chamber using a completely randomized design, with a sufficient distance between them to prevent them from touching each other. Plants were grown in an indoor growth chamber fitted with 12, 400 W HPS horticultural lights (Osram), providing a light intensity of 250 ± 50 μmol/m^2^/s PAR at canopy level. Environmental parameters were kept constant with a temperature of 25°C during the day and 21°C during the night, while RH was set to 70% and photoperiod was set to 18 h/6 h light/darkness. Seeds were sterilized with 0.05% NaClO solution for 3 min and were subsequently rinsed three times with sterile deionized water and placed in Petri dishes lined with a moist paper filter. After 72 h, germinated seeds were transferred to Rockwool plugs (Rockwool) and moistened with a hydroponic solution (Dutchpro Nutrients). The commercial nutrient composition consisted of 4.2% NO_3_‐N, 0.2% NH_4_‐N, 2.3% P_2_O_5_, 6.3% K_2_O, 3.1% CaO, 1.2% MgO, 2.7% SO_3_, 0.6% Na_2_O, 0.3% Cl, plus traces of B 0.006%, Mn 0.015%, Fe 0.018%, Zn 0.006%, Cu 0.002%, Mo 0.002%. The plant nutrient solution was prepared by adding A and B of the vegetative hydroponic stock solution (Dutchpro) to reverse osmosis deionized water, resulting in an electric conductivity (EC) of 0.4 mS/cm. Then, the pH was adjusted to 5.8 with KOH. During the vegetative phase, plants were fertigated once a day until the substrate was completely saturated with the vegetative hydroponic solution A and B (Dutchpro), supplemented with MgSO_4_ and CaCl_2_ to a final concentration of 0.127 and 0.05 g/L, respectively. The EC was increased gradually as the plants grew to reach 0.8 m S/cm while the surplus solution released by the Rockwool plugs was drained.

Three weeks after germination, plants were transplanted into 1‐L Rockwool cubes (Rockwool) fitted with an automated irrigation system, which fertigated the plants three times a day, during the daytime, with circa 150 mL of hydroponic solution. On the same day, the photoperiod was changed to short daylight (12/12 h light/darkness) to induce flowering. As plants started to develop flower buds, the flowering solutions A and B (Dutchpro), supplemented with MgSO_4_ and CaCl_2_, as stated above, were provided until harvest. The EC value was gradually increased until reaching 1.6 m S/cm as plants entered the fourth week of flowering. The nutrient solution concentration was kept constant until harvest. After 7 weeks of flowering, plants were harvested and subsequently dried in darkness in a climate chamber at 20°C and 30% RH for 7 days. In total, 40 plants were sown per cultivar [(4 PGPR + 1 control) × 8 replicates, Table S1]. Samples of the flowers from each plant were taken in equal proportion from the apical, middle and lower flowers and pooled to account for possible variability in specialized metabolite accumulation over plant height. As a result, a representative 1 g of dried flower material per plant was obtained. Samples were stored at −20°C in sealed 10 mL Falcon tubes, and before chemical analysis, samples were ground with a mortar and pestle until a fine powder was obtained.

### 2.4 Phenotypic Measurements

Apart from the onset of flowering, all phenotypic measurements were conducted at the final harvest, 70 days after germination. All plants were measured on the same day. For the onset of flowering, plants were scored visually for flower bud primordia at the third week of flowering with a score of 1 to 3, with the score 3 reflecting the most developed flower primordia. Plant height and number of nodes were measured just before plant harvest. Height measurements were taken from the base of the stem to the apical meristem. Total dry biomass was measured after plants were harvested and dried in darkness in a climate chamber at 20°C and 30% RH for 1 week. The dry weight of the flowers was measured after carefully stripping them from the plant. The harvest index was calculated as flower dry weight divided by total plant dry weight.

### 2.5 Bacterial Colonization

Samples of fresh roots were collected during the final harvest. Root samples were surface‐sterilized with 0.05% NaClO and rinsed with sterile water. Under the laminar flow hood, following aseptic techniques, surface‐sterilized roots were placed in sterile tubes containing sterile water and metal beads. The tubes were shaken to disrupt the plant tissue with TissueLyser (Qiagen), and the lysate was plated on selective media containing the antibiotic combination to which the studied bacteria are resistant. Confirmation of their presence inside the root tissue was carried out visually by comparing the colonies' phenotypic appearance with the known characteristics of the cultured bacteria under study.

### 2.6 Cannabinoid Measurements

Cannabinoids were extracted with 1 mL of methanol from 10 mg of the ground flower material. Samples were sonicated for 20 min in a water bath at room temperature. Subsequently, the tubes were centrifuged at 13000 × *g* to pellet plant material. The supernatant was filtered through a 20 μm RC Minisart filter (Sartorius AG). The extract was kept in a sealed dark glass vial and stored at −20°C until analysis. Cannabinoids were quantified following a modified protocol by Gul et al. (2015) with a reversed‐phase HPLC (Agilent 1200 chromatographic system) equipped with a UV‐photodiode array detector (UV‐DAD) and an auto‐sampler. The separation was achieved on a Luna‐C18 column (Phenomenex). The mobile phase consisted of solvent A 0.1% (v/v) formic acid in water and solvent B 0.1% (v/v) formic acid in acetonitrile. The flow rate was set at 1.2 mL/min. Solvent B was initially set at 30%; after 3 min, it was increased to 75%. At 15 min from the program's start, solvent B was increased to 100% and held for 3 min, then decreased to 30% in the final 2 min of the sample run. The auto‐sampler automatically injected 5 μL of the sample. The DAD detected light absorption at 228, 230, 280, 320, and 360 nm. The wavelength chosen for quantification was 320 nm. Quantification was performed with standards THCA, CBDA, CBGA, Δ^9^‐THC, CBG, and CBD (Merck). A fivefold serial dilution of the standards was performed on the range of quantification, and linear regression in RStudio software (version 4.3.0) was performed. The linearity (*r* ^*2*^) of all calibration regressions was above 0.99.

### 2.7 Statistical Analysis

Principal component analysis (PCA) was performed on the whole dataset and on the phenotypical and chemical data separately. The PCA was scaled and centered. PC1 and PC2 were used to visualize the data.

Two‐way ANOVAs were performed with cultivar and bacterial treatment as fixed factors and total cannabinoids, THCA, Δ^9^‐THC, CBDA, CBGA, and the ratio between the precursor CBGA and the product THCA, as well as CBGA/CBDA and THCA/CBDA, as dependent variables. The ratios were analyzed on the acidic cannabinoids to separate the treatments' effect on the cannabinoid's metabolism from the possible in vivo or post‐harvest degradation.

The effect of cultivar and bacteria on possible in vivo or post‐harvest degradation was assessed with a two‐way ANOVA on the THCA/THC ratio, with cultivar and bacterial treatment as fixed factors and THCA/THC ratio as dependent variables.

The two‐way ANOVA was repeated on the same dependent variables with the different bacterial taxa pooled to have inoculation as a factor to detect possible effects of the inoculation regardless of the bacterial taxa.

Phenotypic variables were analyzed by two‐way ANOVAs with cultivar and bacterial treatment as fixed factors and total plant dry weight, flower dry weight, harvest index, and height as dependent variables. When the normality of residuals and homoscedasticity assumptions were not met, data were Box‐Cox transformed. For count data of flowering initiation and number of nodes, the GLM function was used. Differences between the means of control and treatments were obtained by performing a Dunnett post hoc test. All statistical analyses were carried out with R version 4.3.0.

## 3 Results

### 3.1 All Bacterial Strains Colonized *C. sativa* Roots

The root colonization of all four bacterial strains was confirmed via endophyte‐specific assays. All the root samples analyzed contained the inoculated bacteria, confirming their ability to penetrate within the root tissues of *C. sativa* .

### 3.2 Plant Height and Cannabinoid Profiles Drive Multivariate Trait Separation

The unsupervised Principal component analysis (PCA) on phenotypical and chemical variables combined explained 37.59% and 19.37% of the observed variance on the first two principal components. The traits having influence on the separation of the datapoints were THCA and the harvest index for PC1 and CBGA, CBGA/THCA and height for PC2. To further investigate the dataset, data points were colored based on cultivars (Figure S1), inoculation (Figure S1B), and bacterial taxa with which they were treated (Figure S1C). This showed that the separation between the cultivars was driven mainly by the height of the plants, the harvest index, and the cannabinoid content, with CBGA, THCA, CBDA, and the ratio CBGA/THCA and THCA/CBDA having a major influence on the separation.

Interestingly, the inoculation also resulted in clustering. Although the control samples were mixed with the inoculated samples, they were mainly located in the positive quadrant of the PC2. In this case, the separation was mainly driven by plant height, plant and flower biomass as well as CBGA, CBDA, THCA, and their respective ratios.

### 3.3 Phenotypic Traits Differ by Cultivar With no Influence of Bacteria or Inoculation

**FIGURE 1:**
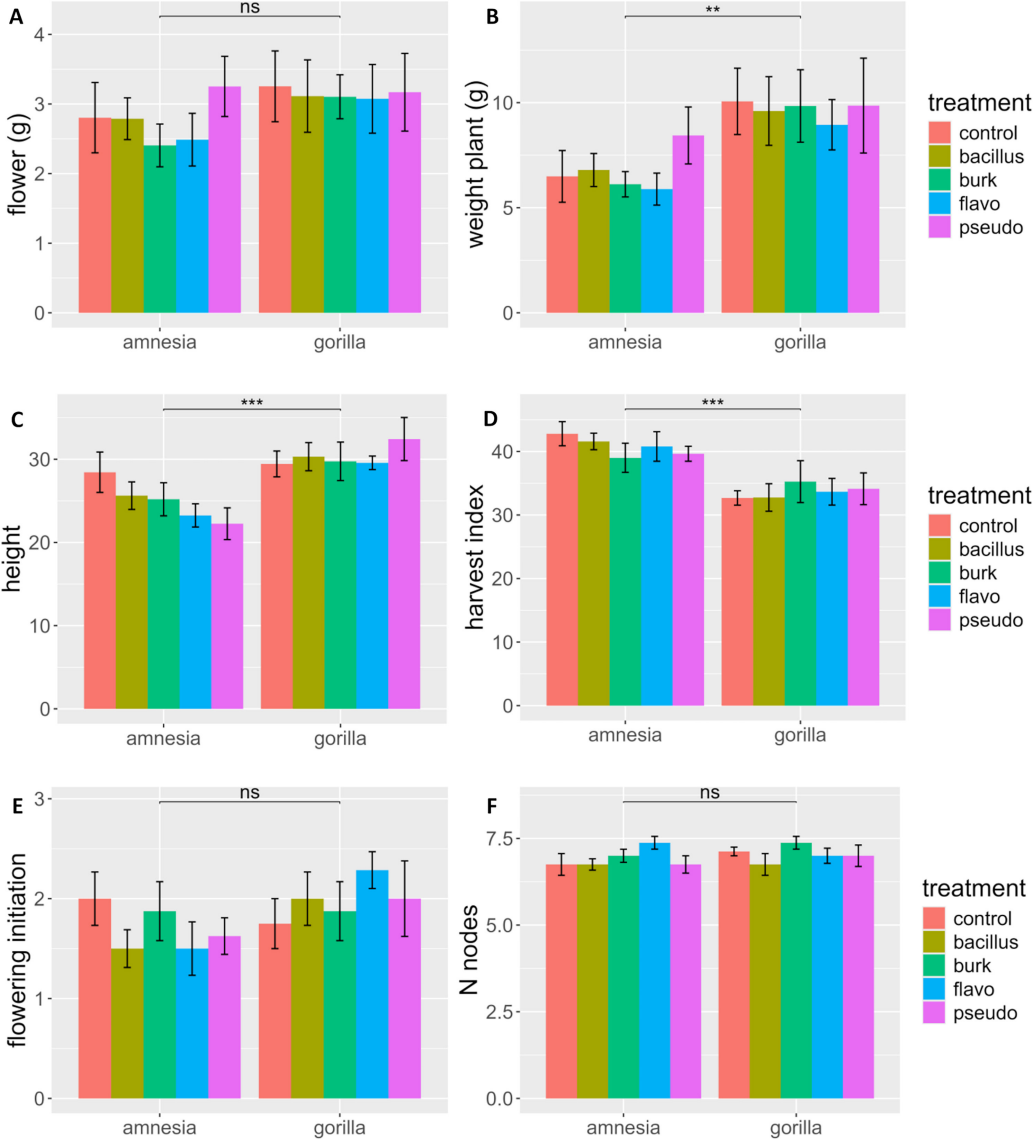
Phenotypical variables of the *C. sativa* cultivars Amnesia and Gorilla inoculated with four different PGPR bacterial taxa and a control mock solution, *Bacillus* (Bacillus), *Burkholderia* (Burk), *Flavobacterium* (Flavo), and *Pseudomonas* (Pseudo). (A) dry flower weight, (B) dry plant weight, (C) height, (D) harvest index, (E) flowering initiation, (F) number of nodes. Bars represent means ± standard errors. Dry plant weight was logarithmically transformed as indicated by the Box‐Cox function. Different symbols above bars indicate significant differences at *p < 0.05, **p < 0.01, ***p < 0.001 and “ns” *p* > 0.05. Analysis was performed on 79 individuals. ANOVA dry plant weight: Cultivar *F*_1,69_ = 9.477, *p* = 0.003; Bacteria *F*_4,69_ = 0.286, *p* = 0.886; Interaction *F*_4,69_ = 0.457, *p* = 0.767. ANOVA plant height: Cultivar *F*_1,69_ = 19.138, *p* = 4.22e−05; Bacteria *F*_4,69_ = 0.525, *p* = 0.717; Interaction *F*_4,69_ = 1.346, *p* = 0.262. ANOVA harvest index: Cultivar *F*_1,69_ = 28.504, *p* = 1.13e−06; Bacteria *F*_4,69_ = 0.040, *p* = 0.997; Interaction *F*_4,69_ = 0.768, *p* = 0.550.

For total plant dry weight, harvest index, and plant height a significant effect of the cultivar was detected, with Gorilla being a taller (30.18 ± 5.02 cm) and a heavier (9.59 ± 4.43 g) cultivar than Amnesia (24.95 ± 5.55 cm) (6.75 ± 2.81 g) but presenting a lower harvest index (33.73 ± 6.13) compared to Amnesia (40.76 ± 5.17) (Figure 1, Table S2). No significant effects were detected for inoculation or bacterial strain.

### 3.4 Cultivar Determines Cannabinoid Levels While Bacterial Inoculation Shifts Cannabinoid Metabolism

**FIGURE 2:**
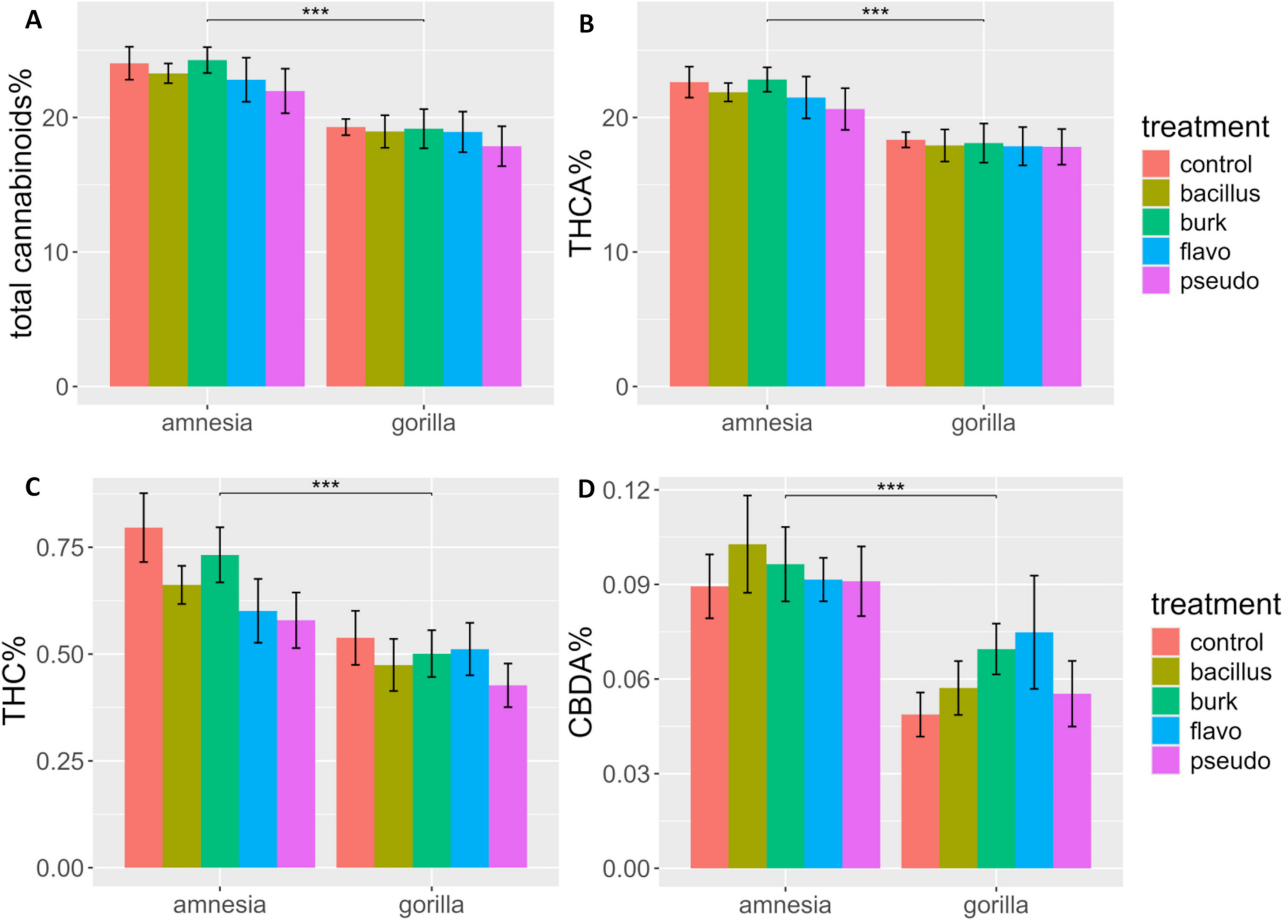
Bar plot of (A) total cannabinoid%, (B) THCA%, (C) THC%, and (D) CBDA% of flower dry weight of an HPLC analysis of *Cannabis sativa* L. cultivars Amnesia and Gorilla, treated with *Bacillus* (Bacillus), *Burkholderia* (Burk), *Flavobacterium* (Flavo), and *Pseudomonas* (Pseudo). Bars represent means ± standard errors. A logarithmic transformation was performed for CBDA% as indicated by a Box‐Cox test. Analysis was performed on 79 individuals. For CBDA% on 78 individuals. For results of two‐way ANOVA, see Table 1. Symbols above bars “***” indicate significant differences at *p* < 0.001.

The cultivar Amnesia contained a significantly higher total cannabinoid content (23.27% ± 3.57%) than Gorilla (18.84% ± 3.44%) (Figure 2A, Table 1A).

**TABLE 1:** *F* and *p*‐values of ANOVA test on cannabinoids from two *C. sativa* cultivars (A) inoculated with one of four different bacterial strains (Bacteria) or a control mock solution and (B) bacterial taxa pooled and analyzed as inoculum (Inoculum) compared to a control mock solution.

A Test: bacterial taxa	Cultivar		Bacteria		Cultivar × bacteria	
	*F*_1,69_	*p*	*F*_4,69_	*p*	*F*_4,69_	*p*
Total Cannabinoids%	29.452	**8.01e−07**	0.649	0.629	0.072	0.990
THCA%	26.863	**2.07e−06**	0.634	0.640	0.070	0.991
Log (CBDA%)	27.229	**1.86e−06**	0.664	0.619	0.568	0.687
Log (CBGA%)	23.090	**8.7e−06**	2.860	**0.03**	0.595	0.668
THC%	21.395	**1.7e−05**	1.997	0.105	0.545	0.703
Log (CBGA/THCA)	1.487	0.227	2.857	**0.03**	0.558	0.694
CBGA/CBDA	2.421	0.124	0.253	0.907	0.418	0.795
√(THCA/CBDA)	13.566	**4.58e−04**	1.461	0.224	0.842	0.503
(THCA/THC)^−1^	4.023	**0.0488**	1.754	0.1482	0.408	0.8021

B Test: bacterial inoculation	Cultivar		Inoculum		Cultivar × inoculum	
	*F*_1,75_	*p*	*F*_1,75_	*p*	*F*_1,75_	*p*
Total Cannabinoids%	30.983	**3.85e−07**	0.584	0.447	0.038	0.846
THCA%	28.316	**1.03e−06**	0.677	0.413	0.024	0.878
Log(CBDA%)	28.850	**8.67e−07**	2.292	0.134	0.977	0.326
Log(CBGA%)	24.082	**5.24e−06**	10.977	**0.001**	0.401	0.528
THC%	21.778	**1.31e−05**	4.710	**0.033**	0.875	0.353
Log(CBGA/THCA)	1.569	0.214	11.899	**0.001**	0.361	0.55
CBGA/CBDA	2.558	0.114	0.552	0.460	0.131	0.718
√(THCA/CBDA)	14.195	**3.29e−04**	5.365	**0.023**	1.424	0.237
(THCA/THC)^−1^	4.134	**0.0456**	4.109	**0.0462**	0.696	0.4066

*Note:* Significant values are highlighted in bold for *p*‐value ≤ 0.05. When assumptions on homoscedasticity and normality of the residuals were not met, variables were square‐rooted, logarithmically transformed or inverted as suggested by the Box‐Cox function. Analysis was performed on 79 individuals. For CBDA%, CBGA/CBDA and THCA/CBDA on 78 individuals, as CBDA concentration was under the detection limit for one sample, and the data point could not be logarithmically transformed or be the denominator in a fraction.

The main compound (THCA) followed the same trend as the total cannabinoids (Figure 2B), accounting for 94.6% and 94.05% of the total cannabinoid fraction for Gorilla and Amnesia, respectively. A significant effect was detected for cultivar, with Amnesia producing more THCA (21.89% ± 3.3%) than Gorilla (17.84% ± 3.34%) (Table 1A). The bacterial treatment and the interaction between the cultivar and bacteria were not significant.

Measurements on Δ^9^‐THC showed the same trend observed for THCA (Figure 2C), with cultivar Amnesia showing significantly higher Δ^9^‐THC concentrations (0.67% ± 0.20%) than Gorilla (0.49% ± 0.16%). The bacterial treatment and interaction between the bacteria and cultivar were not significant (Table 1A).

Measurements on Δ^9^‐THC also served the purpose of assessing the decarboxylation of the samples. With a mean value of 0.58% of the dry flower weight, the Δ^9^‐THC content was low. On average, Δ^9^‐THC was 2.73% ± 0.63% of the total cannabinoid fraction, a low value considering that samples were not flash‐frozen and freeze‐dried. Moreover, the other decarboxylated cannabinoids, CBD and CBG, were found to be under the detection limit for all samples, confirming the low level of sample degradation. Overall, cannabinoid degradation was not affected by the different bacterial taxa. On the other hand, the effect of the cultivar was significant (Table 1A). In this case, the THCA/THC ratio of Amnesia (34.22 ± 7.25) was significantly lower compared to Gorilla (39.19 ± 11.93), reflecting a higher degradation rate in Amnesia.

Analysis of CBDA concentration (Figure 2D) showed that Amnesia (0.094% ± 0.031%) contained a significantly higher concentration than Gorilla (0.061% ± 0.030%) (Table 1A). The bacterial treatment and the interaction were not significant.

**FIGURE 3:**
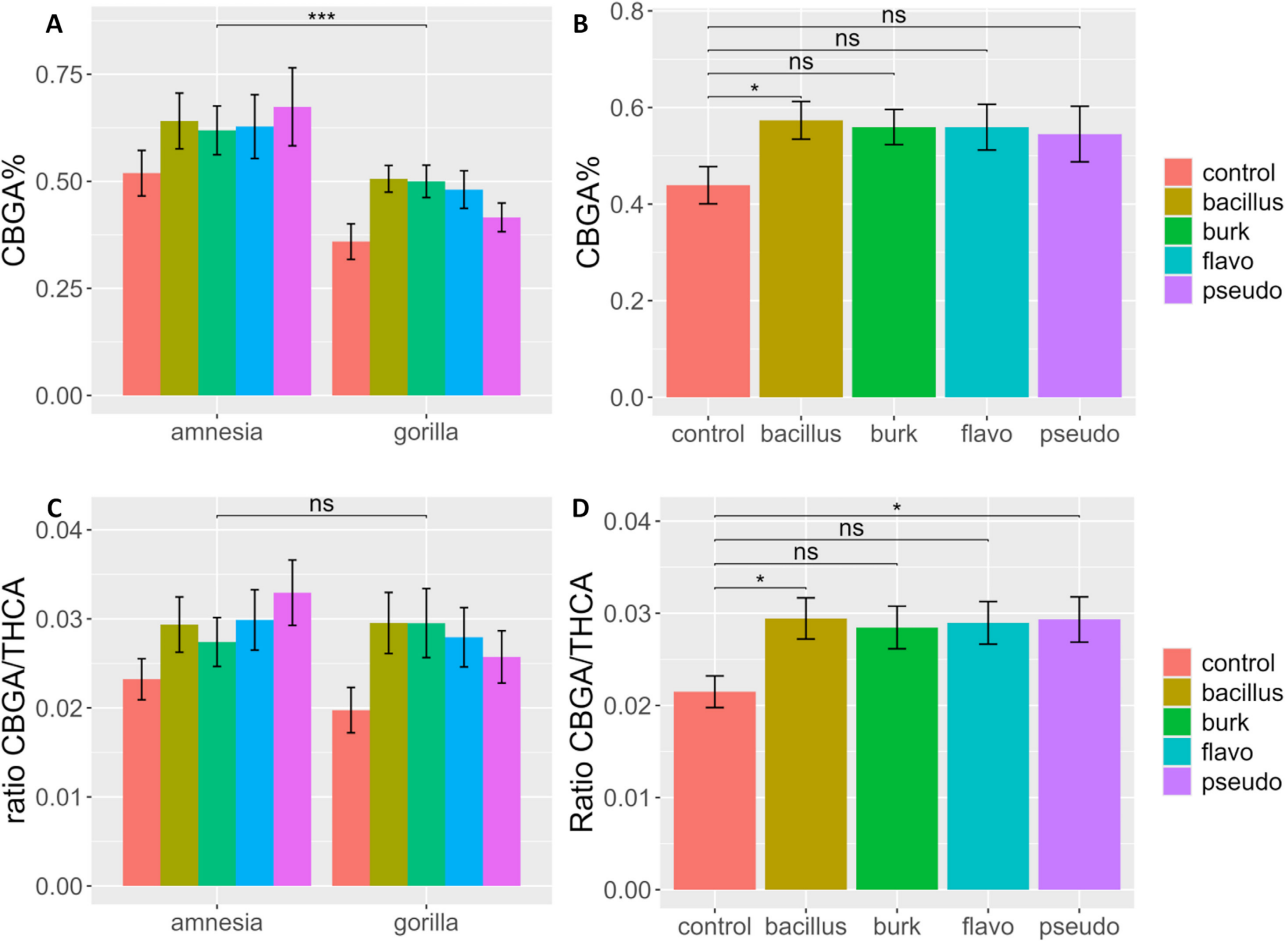
Bar plot of (A and B) CBGA% and (C and D) CBGA/THCA ratio of the flower dry weight of an HPLC analysis of flowers of *Cannabis sativa* L. cultivars Amnesia and Gorilla treated with *Bacillus* (Bacillus), *Burkholderia* (Burk), *Flavobacterium* (Flavo), and *Pseudomonas* (Pseudo). Bars represent means ± standard errors. A logarithmic transformation was performed as indicated by a Box‐Cox test for both variables. Analysis was performed on 79 individuals. For results of two‐way ANOVA, see Table 1. Different symbols above bars indicate significant differences at *p < 0.05, **p < 0.01, ***p < 0.001, and “ns” *p* > 0.05. The B and D panels display the CBGA concentration and CBGA/THCA ratio for the bacterial treatments when treatments were tested against the control via post hoc Dunnett test.

CBGA concentrations revealed an interesting pattern (Figure 3A,B). For this precursor cannabinoid, a significant effect of the bacterial treatment and cultivar was detected. The post hoc test detected a significant increase of +30.6% when plants were inoculated with *Bacillus* compared to the control (*p* = 0.036) (Table 1A). Amnesia appears to be a better producer, with a concentration of CBGA significantly higher (0.62% ± 0.19%) than Gorilla (0.45% ± 0.12%).

Analysis of the ratios between precursor and products (CBGA/THCA, CBGA/CBDA), as well as both products (THCA/CBDA), revealed significant differences (Table 1A). These ratios were analyzed to put the major cannabinoids in relation and better understand the metabolic changes induced by the bacteria. For THCA/CBDA, the cultivar effect was significant, with Gorilla having a higher ratio than Amnesia, reflecting the higher content of THCA compared to CBDA in this cultivar.

The CBGA/THCA ratio instead shifted significantly toward higher concentrations of the precursor CBGA to the detriment of THCA when plants were inoculated with the different bacterial strains (Table 1A; Figure 3C,D). A significant increase in the ratio of +37.06% (*p* = 0.044) and +36.52% (*p* = 0.048) was detected when plants were inoculated with *Bacillus* and *Pseudomonas*, respectively. Interestingly, the effects of cultivar and the interaction between cultivar and bacteria were not significant. Altogether, the CBGA/THCA ratio data show that cultivars reacted similarly and that the different bacterial taxa induced the same type of changes in the cannabinoid metabolism.

**FIGURE 4:**
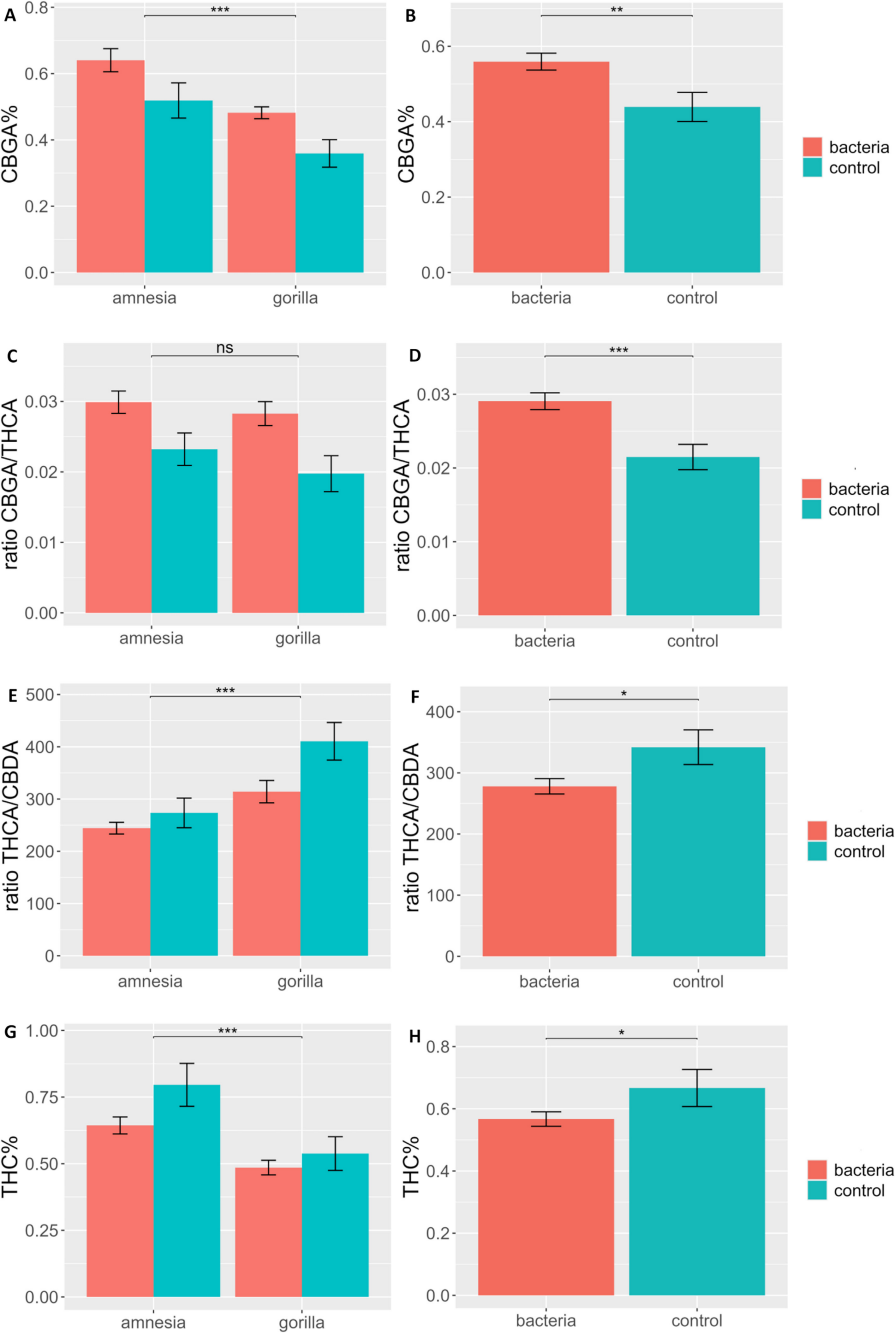
Bar plot of (A and B) CBGA%, (C and D) CBGA/THCA ratio, (E and F) THCA/CBDA ratio and (G and H) Δ^9^‐THC% of the flower dry weight of an HPLC analysis of flowers of *Cannabis sativa* L. cultivars Amnesia and Gorilla treated with the bacterial inoculum and compared to a control mock inoculation. Bars represent means ± standard errors. A logarithmic transformation was performed for CBGA%, CBGA/THCA, while a square root transformation was performed for the THCA/CBDA ratio, as indicated by a Box‐Cox test. Analysis was performed on 79 individuals for THCA/CBDA on 78 individuals. For results of the two‐way ANOVA, see Table 1. Different symbols above bars indicate significant differences at *p < 0.05, **p < 0.01, ***p < 0.001 and “ns” *p* > 0.05. Panels B, D, F and H display the CBGA, THC concentrations and the CBGA/THCA, THCA/CBDA ratios for the inoculation.

Inoculation, regardless of bacterial strain, resulted in significant changes in concentrations of CBGA, Δ^9^‐THC, and the ratios of CBGA/THCA, THCA/CBDA and THCA/THC (Table 1B). In inoculated plants, the concentration of CBGA increased significantly by +27.37% (Figure 4B), while the concentration of Δ^9^‐THC decreased significantly (−15.76%) (Figure 4H) when compared to controls. The THCA/THC ratio revealed a significant effect of the inoculation, with the treated plants having a significantly higher ratio (37.44 ± 9.97) than control plants (33.63 ± 10.33), indicating that the bacteria (regardless of the taxa) resulted in less in vivo and post‐harvest decarboxylation of the main cannabinoid THCA. In the case of the major cannabinoid ratios, the CBGA/THCA followed the same trend as previously observed with a significant increase by +35.23% toward higher concentrations of the precursor CBGA to the detriment of THCA when plants were inoculated with PGPRs (Figure 4D). Surprisingly, the ratio of THCA/CBDA was also found to be significant with a shift of −18.70% compared to control when plants were inoculated with PGPRs (Figure 4F). Indeed, although not significantly, the concentration of THCA was found to decrease by −3.65%, while CBDA increased by +15.69%. In combination, these changes resulted in a significant shift of the ratio toward higher concentrations of CBDA at the cost of THCA.

## 4 Discussion

This experiment showed that all four bacterial genera survived the rhizospheric hydroponic environment and were able to successfully penetrate the roots and colonize the plant tissue, despite the hydroponic environment being very different from the soil. This has important implications regarding their applications in agricultural hydroponic settings.

Interestingly, the inoculation with PGPRs did not cause statistically relevant changes in the *C. sativa* phenotype. This demonstrates that PGPRs' colonization did not negatively affect the *C. sativa* cultivars under study.

On the other hand, measurements on the cannabinoid concentrations revealed that bacteria colonization could influence the cannabinoid metabolism, potentially in beneficial ways. A closer look at the main cannabinoid via the THCA/THC ratio analysis revealed a significant effect of the PGPRs. The bacterial inoculation, regardless of taxa, significantly decreased the in vivo and post‐harvest decarboxylation of the main cannabinoid THCA into Δ^9^‐THC. These findings highlight that PGPRs may have a beneficial effect on cannabinoid accumulation. PGPRs did not negatively affect the total content of cannabinoids, while simultaneously contributing to their protection from decarboxylation, with important implications for the post‐harvest preservation of the acidic chemotype.

The other major cannabinoids were also significantly influenced. The ratio between the precursor CBGA and the product THCA significantly shifted toward higher concentrations of the precursor when plants were inoculated with PGPRs, with *Bacillus* and *Pseudomonas* having the strongest effects. On the other hand, the THCA/CBDA ratio provides information regarding a possible shift between the production of these two metabolites. PGPRs significantly influenced this ratio by shifting it toward higher concentrations of CBDA, at the cost of THCA.

With this research, we showed that PGPRs can induce metabolic changes in drug‐type *C. sativa* that block the production of the main cannabinoid THCA while redirecting energies to the production of CBDA, with consequent accumulation of the precursor CBGA. Indeed, in high THCA cultivars, the synthesis of CBDA is carried out by non‐functional CBDA synthases (Weiblen et al. 2015), which are only able to produce tiny quantities of the compound and are probably unable to quickly convert CBGA into CBDA. Further tests are needed to confirm the probable upregulation of non‐functional CBDA synthases and the downregulation of THCA synthases, likely causing the resulting chemotype shift. Overall, our results indicate that the bacteria's colonization can influence the cannabinoid metabolism of drug‐type *C. sativa* . Moreover, these effects influenced *C. sativa* metabolism regardless of the cultivar. Even more interestingly, all the PGPR taxa had the same effect on cannabinoids and their ratios, although with different strengths. For these reasons, the results have broad applicability and point to a common mechanism by which PGPRs can influence *C. sativa'*s specialized metabolism.

In this regard, by ruling out the effect of PGPRs on nutrient availability, the results suggest that bacterial colonization directly induces the observed changes in cannabinoid metabolism, probably by inducing ISR or through signal molecules. Indeed, a recent study demonstrated how *Bacillus* is able to influence *C. sativa* phenotype through the production of phytohormones, like indole‐3‐acetic acid (IAA), triggering genes involved in the biosynthesis of phenylpropanoid, plant–pathogen interaction, and plant hormone signal transduction pathways (Aunkam et al. 2024).

Additionally, our findings confirm previous speculations (Lyu et al. 2019) and observations (Comeau et al. 2021) on *Pseudomonas* and *Bacillus*. Indeed, in this study, *Pseudomonas* and *Bacillus* were the only two taxa having a significant effect on cannabinoid metabolism.

To the best of our knowledge, this is the first report documenting the effects of PGPRs on cannabinoid metabolism of hydroponically grown high‐THCA drug‐type *C. sativa* cultivars. Moreover, our findings have broad applicability as tests were performed on two distinct cultivars with plants originating from seeds and not clonally propagated genotypes. At the same time, the changes observed are shared across the cultivars and the four bacterial taxa examined in the study.

Although this research shed light on the important effects of PGPRs on *C. sativa* , research in this field is still in its infancy. More research applying genomics, transcriptomics, metabolomics, and phytocannabinomics (Cerrato et al. 2021) could reveal other significant changes induced on *C. sativa* metabolism. Additionally, scaling the design with the use of *Bacillus* and *Pseudomonas* consortia and including CBDA‐dominant, balanced or CBGA chemotypes might have positive outcomes in improving yields and modulating the plants' chemical expression.

The findings and methodology of this research lay the groundwork for further evaluating and exploiting the potential beneficial relationship between PGPRs and *C. sativa* and implementing their sustainable applications in the agricultural, biotechnological, and pharmaceutical sectors.

## Author Contributions

**Francesco Tonolo:** conceptualization, methodology, plant growth, cannabinoid analysis, data acquisition, statistical analysis, formal analysis, investigation, manuscript writing, review and editing. **Bobbie Sewalt:** plant growth, data acquisition. **Klaas Vrieling:** conceptualization, supervision, manuscript writing, review and editing. **Young Hae Choi:** supervising chemical analysis and manuscript review. All authors contributed to the article and approved the submitted version.

## Funding

This work was supported by the Natural Products Laboratory, Institute of Biology, Leiden University, Leiden, Netherlands and Aboveground‐belowground Interaction Group, Plant Cluster, Institute of Biology, Leiden University, Leiden, Netherlands.

## Supporting information


**Data S1:** Supporting Information.

## Data Availability

The raw data that support the findings of this study are available from the corresponding author upon reasonable request.
