# Supplementary material for: Plant Growth‐Promoting Rhizobacteria Colonize Δ9 ‐Tetrahydrocannabinolic Acid Drug‐Type Cannabis sativa L. Roots and Modulate Cannabinoid Metabolism
Source: Physiol Plant. 2026 Jan 24;178(1):e70756. doi: 10.1111/ppl.70756 (PMC12831123; doi:10.1111/ppl.70756)

Plant growth-promoting rhizobacteria colonize Δ^9-tetrahydrocannabinolic acid drug-type *Cannabis sativa* L. roots and modulate cannabinoid metabolism

Francesco Tonolo[1]*, Bobbie Sewalt[1], Klaas Vrieling[1] and Young Hae Choi[2]*

[1]Aboveground-belowground Interaction Group, Plant Cluster, Institute of Biology, Leiden University, Leiden, Netherlands

[2]Natural Products Laboratory, Institute of Biology, Leiden University, Leiden, Netherlands

Table S1 Number of *C. sativa* plants grown for the experiment, one plant from cultivar Gorilla treatment Flavobacterium was removed because of irrigation failure and resulting drought stress.

Cultivar	Control	Pseudomonas	Flavobacterium	Bacillus	Burkholderia
Amnesia	8	8	8	8	8
Gorilla	8	8	7	8	8

Table S2 Phenotypical variables of the *C. sativa* cultivars Amnesia (A) and Gorilla (G) inoculated with four different PGPR bacterial taxa and a control mock solution. Values are means ± standard deviations. Dry plant weight was logarithmically transformed as indicated by the Box-Cox function.

Treatment	Dry plant weight (g)		Dry flower weight (g)		Plant height (cm)		Number of nodes		Flowering initiation (visual score 1 to 3)		Harvest index (%)	
	A	G	A	G	A	G	A	G	A	G	A	G
Control	6.5±3.5	10.1±4.5	2.8±1.4	3.3±1.4	28.4±6.9	29.4±4.4	6.8±0.9	7.1±0.4	2.0±0.8	1.8±0.7	42.8±5.4	32.6±3.2
Bacillus	6.8±2.2	9.6±4.6	2.8±0.9	3.1±1.5	25.6±4.7	30.3±4.8	6.8±0.5	6.8±0.9	1.5±0.5	2.0±0.8	41.6±3.7	32.8±6.1
Burkholderia	6.1±1.7	9.8±4.9	2.4±0.9	3.1±0.9	25.2±5.6	29.8±6.5	7.0±0.5	7.4±0.5	1.9±0.8	1.9±0.8	39.0±6.5	35.3±9.3
Flavobacterium	5.9±2.2	9.0±3.2	2.5±1.1	3.1±1.3	23.3±4.0	29.6±2.2	7.4±0.5	7.0±0.6	1.5±0.8	2.3±0.5	40.8± 6.6	33.7±5.5
Pseudomonas	8.4±3.8	9.4±5.7	3.3±1.2	3.1±1.4	22.3±5.4	31.8±6.6	6.8±0.7	6.9±0.8	1.6±0.5	2.0±0.9	39.6±3.3	34.3±6.1

Fig. S1 Principal component analysis of 4 phenotypical and 8 chemical variables. Each point indicates an individual genotype. Observations were colored by (**A**) cultivar, (**B**) inoculation (pool of PGPRs) and (**C**) bacterial taxa, *Bacillus* (Bacillus), *Burkholderia* (Burk), *Flavobacterium* (Flavo) and *Pseudomonas* (Pseudo). Arrows indicate the PCA loading, showing how much each original variable contributes to the PCA.

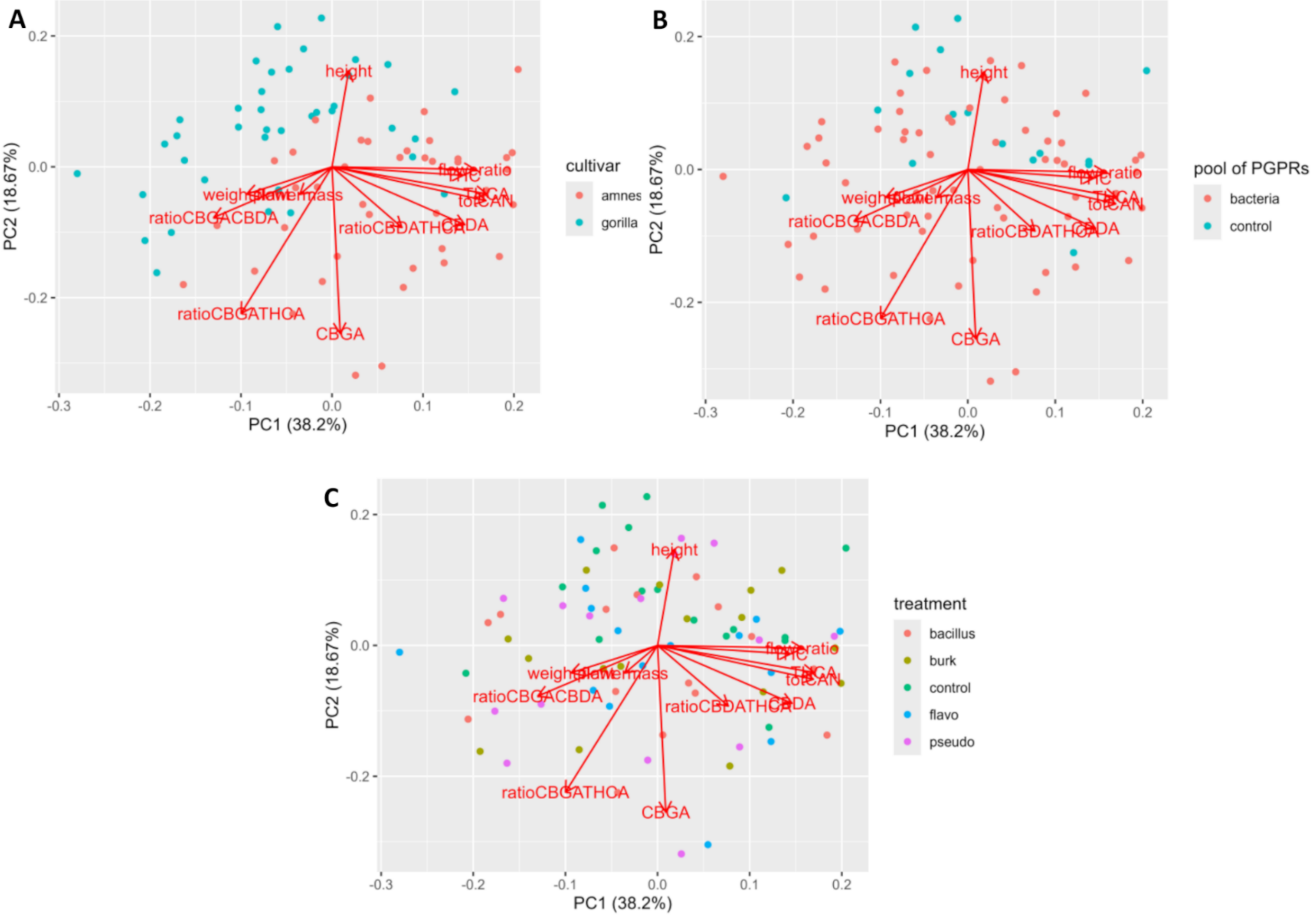

Supplement: Supplementary file 1 — Data S1: Supporting Information. [file PPL-178-e70756-s001.pdf]
